# Heterogeneous Diffusion and Nonlinear Advection in a One-Dimensional Fisher-KPP Problem

**DOI:** 10.3390/e24070915

**Published:** 2022-06-30

**Authors:** José Luis Díaz Palencia, Saeed ur Rahman, Antonio Naranjo Redondo

**Affiliations:** 1 Department of Mathematics and Education, Universidad a Distancia de Madrid, 28400 Madrid, Spain; 2 Technology Programs, Schiller International University, Calle Serrano 156, 28002 Madrid, Spain; antonio.naranjo@schiller.edu; 3 Department of Mathematics, COMSATS University Islamabad, Abbottabad 22060, Pakistan; saeed@cuiatd.edu.pk

**Keywords:** higher-order diffusion, Fisher-KPP problem, instabilities, existence, uniqueness, asymptotic, positivity, 35K92, 35K91, 35K55

## Abstract

The goal of this study is to provide an analysis of a Fisher-KPP non-linear reaction problem with a higher-order diffusion and a non-linear advection. We study the existence and uniqueness of solutions together with asymptotic solutions and positivity conditions. We show the existence of instabilities based on a shooting method approach. Afterwards, we study the existence and uniqueness of solutions as an abstract evolution of a bounded continuous single parametric (*t*) semigroup. Asymptotic solutions based on a Hamilton–Jacobi equation are then analyzed. Finally, the conditions required to ensure a comparison principle are explored supported by the existence of a positive maximal kernel.

## 1. Introduction and Problem Outline

The problem is formulated as follows:

(1) $$w_{t}=-w_{xxxx}+c{\left(w^{q}\right)}_{x}+w\left(a-w\right),$$

with the initial conditions:

(2) $$\begin{array}{c} w_{0}\left(x\right)\in X=L_{loc}^{2}\left(R\right)\cap L^{\infty }\left(R\right),\\ w_{0,x}\in L^{q}\left(R\right), \end{array}$$

where q>0,a>0.

The interactive motion in a domain requires studying the diffusion accurately, for example, via statistical concepts based on a random walk approach (see [1]). Other proposals to analyze diffusive processes have been followed in [2,3] based on the free energy of Landau–Ginzburg. This approach ends in a non-regular (or heterogeneous) diffusion compared to that derive from the classical Fick law. Such non-regularity can be observed as oscillating profiles of solutions in the proximity of two particular concentration values given by the stationary conditions.

Reaction–diffusion models were formally introduced by Fisher [4] and Kolmogorov, Petrovskii and Piskunov [5] to study the interaction of genes and the behaviour of flames in combustion theory, respectively. The approach followed by the cited authors was based on a Fickian diffusion and a reaction term of the following form: f(u)=u(1−u). The problem was tracked with the Travelling Waves (TW) solutions to understand the behaviour of diffusion acting in a wave front and to understand the propagation features of each specie involved. The Fisher or KPP model is ubiquitous to several applied sciences (see for example [6,7,8]). Furthermore, and more recently, KPP-models have been analyzed with fractional operators [9], with a p-Laplacian Porous Medium Equation [10] and higher order operators [11].

Alternatively to the heterogeneous diffusion discussed, the higher-order operators may be seen as perturbations to regular-order two diffusion; see [12,13,14,15] for some extensions of the Fisher–Kolmogorov equation to fourth-order operators.

Some further notable analysis can be cited related to modelling with heterogeneous diffusion. For instance, in [16], the authors study a biological interaction between species with advection, which precludes a non-linear diffusion. In addition, [17] studies the spectral stability of a class of solutions to model the haptotaxis cancer invasion.

The proposed equation is formed of a higher-order operator (in the sense of non-homogeneous), a non-linear advection and a KPP term:

(3) $$\begin{array}{c} w_{t}=-w_{xxxx}+c{\left(w^{q}\right)}_{x}+w\left(a-w\right),\\ w_{0}\left(x\right)\in X=L^{\infty }\left(R\right)\cap L_{loc}^{2}\left(R\right),\;\;\;\;w_{0,x}\in L^{q}\left(R\right),\;\;\;\;a>0,\;\;\;\;\;q>0. \end{array}$$

The paper layout is as follows:

Firstly, solutions are proved to exhibit oscillations supported by a shooting method. Afterwards, we study the existence of solutions as an abstract evolution of a bounded continuous single parametric (*t*) operator. We continue with the study of uniqueness and a precise evolution of such unique solution is obtained under the Hamilton–Jacobi equation scope. Finally, the conditions required to ensure a comparison principle are explored.

## 2. Existence and Uniqueness

For the analysis of existence and uniqueness, we consider the following norm:

(4) $${∥w∥}_{\Gamma }^{2}=\int_{R}\Gamma \left(\omega \right)\sum_{k=0}^{4}{\left|D^{k}w\left(\omega \right)\right|}^{2}d\omega ,$$

where $D=\frac{d}{d\omega }$, $w\in H_{\Gamma }^{4}\left(R\right)\subset L_{\Gamma }^{2}\left(R\right)\subset L^{2}\left(R\right)$ and the weight Γ is considered as (see [11] together with [18]):

(5) $$\Gamma \left(\omega \right)=e^{a_{0}{\left|\omega \right|}^{\frac{4}{3}}-\frac{1}{\omega^{q}}\frac{1}{t^{\gamma }}\int_{0}^{t}\left({∥w_{x}\left(s\right)∥}^{q}+1\right)ds},$$

$a_{0}>0$ sufficiently small and γ>q+1.

The defined functional space form of functions $w\in H_{\Gamma }^{4}\left(R\right)\subset L_{\Gamma }^{2}\left(R\right)\subset L^{2}\left(R\right)$ with norm ${∥w∥}_{\Gamma }$ is a complete Banach space. This last statement follows from standard theory: Consider a sequence $\left\{w_{n}\left(\omega \right):n\in N\right\}\in H_{\Gamma }^{4}$. To this end, fix ε≥0 and assume the Cauchy definition; that is, there shall exist μ∈N such that given m,n>μ, ${∥w_{m}-w_{n}∥}_{\Gamma }\leq \varepsilon$.

### 2.1. A Priori Bounds

Let us denote by $L=(-D_{x}^{4}+qw^{q-1}cD_{x})$ the spatial operator and assume the homogeneous equation:

(6) $$w_{t}=Lw.$$

Then, the following lemma holds for different conditions in the initial distribution (Note that these conditions are different to that in (3), but are described to further characterize the bounds of solutions).

**Lemma** **1.** *Given $w_{0}\in L^{2}\left(R\right)$, then:*

(7) $${∥w∥}_{L^{2}}\leq {∥w_{0}∥}_{L^{2}}.$$

*Let us consider $r\in R^{+}$, so that given $w_{0}\in H^{r}\left(R\right)\cap L^{2}\left(R\right)$:*

(8) $${∥w∥}_{H^{r}}\leq {∥w_{0}∥}_{H^{r}},$$

*and*

(9) $${∥w∥}_{H^{r}}\leq {∥w_{0}∥}_{L^{2}},\;\;\;\;\;for\;\;\;t\geq \frac{r}{4}.$$

*In addition,*

(10) $${∥w∥}_{\Gamma }\leq \kappa {∥w∥}_{H^{r}}\leq \kappa {∥w_{0}∥}_{H^{r}},\;\;\;\;\;\;\;\kappa =25\;\underset{\zeta \in R}{\sup}\left\{w,D^{1}w,D^{2}w,D^{3}w,D^{4}w\right\}.$$

**Proof.**  The fundamental solution to the basic evolution equation can be expressed as:

(11) $$w\left(x,t\right)=e^{tL}w_{0}\left(x\right),$$

and considering the Fourier transformed function in (ζ):

(12) $$\hat{w}\left(\zeta ,t\right)=e^{t(-\zeta^{4}+q{\hat{w}}^{q-1}c\zeta i)}\;{\hat{w}}_{0}\left(\zeta \right).$$

Let us consider now the isometric Fourier property in $L^{2}$:

(13) $$\begin{array}{cc} {∥w∥}_{L^{2}}^{2} & =\int_{-\infty }^{\infty }|e^{t2(-\zeta^{4}+q{\hat{w}}^{q-1}c\zeta i)}\left|\;\right|{\hat{w}}_{0}\left(\zeta \right){|}^{2}d\zeta =\int_{-\infty }^{\infty }e^{-2\zeta^{4}t}{\left|{\hat{w}}_{0}\left(\zeta \right)\right|}^{2}d\zeta \\ & \leq \underset{\zeta \in R}{\sup}\left(e^{-2\zeta^{4}t}\right)\int_{-\infty }^{\infty }{\left|{\hat{w}}_{0}\left(\zeta \right)\right|}^{2}d\zeta ={∥w_{0}∥}_{L^{2}}^{2}. \end{array}$$

Then, ${∥w∥}_{L^{2}}\leq {∥w_{0}∥}_{L^{2}}$. Now, assume the following mollifying norm for $r\in R^{+}$ and 0≤t<∞ satisfying the $A_{p}$-condition (see [19]) for p=1:

(14) $${∥w∥}_{H^{r}}^{2}=\int_{-\infty }^{\infty }e^{r\zeta^{2}}\;{\left|\hat{w}\left(\zeta ,t\right)\right|}^{2}d\zeta .$$

Then:

(15) $$\begin{array}{cc} {∥w∥}_{H^{r}}^{2} & =\int_{-\infty }^{\infty }e^{r\zeta^{2}}|\hat{w}\left(\zeta ,t\right){|}^{2}d\zeta =\int_{-\infty }^{\infty }e^{r\zeta^{2}}|e^{t2(-\zeta^{4}+q{\hat{w}}^{q-1}c\zeta i)}\left|\;\right|{\hat{w}}_{0}\left(\zeta \right){|}^{2}d\zeta \\ & \leq \underset{\zeta \in R}{\sup}\left(e^{-2\zeta^{4}t}\right)\int_{-\infty }^{\infty }e^{r\zeta^{2}}{\left|{\hat{w}}_{0}\left(\zeta \right)\right|}^{2}d\zeta ={∥w_{0}∥}_{H^{r}}^{2}. \end{array}$$

Assume $w_{0}\in L^{2}\left(R\right)$, then:

(16) $${∥w∥}_{H^{r}}^{2}=\int_{-\infty }^{\infty }e^{r\zeta^{2}}|\hat{w}\left(\zeta ,t\right){|}^{2}d\zeta \leq \underset{\zeta \in R}{\sup}\left(e^{r\zeta^{2}}\;e^{-2\zeta^{4}t}\right)\int_{-\infty }^{\infty }{\left|{\hat{w}}_{0}\left(\zeta \right)\right|}^{2}d\zeta .$$

After a simple operation, the following holds:

(17) $${∥w∥}_{H^{r}}^{2}\leq {\left(\frac{r}{4t}\right)}^{1/2}{∥w_{0}∥}_{L^{2}}^{2},\;\;\;\;\;\;{∥w∥}_{H^{r}}\leq {∥w_{0}∥}_{L^{2}},$$

for $t\geq \frac{r}{4}$, as postulated.

Eventually:

(18) $${∥w∥}_{\Gamma }^{2}=\int_{R}\Gamma \left(\zeta \right)\sum_{k=0}^{4}|D^{k}w\left(\zeta \right){|}^{2}d\zeta \leq \int_{R}e^{r\zeta^{2}}\sum_{k=0}^{4}|D^{k}w\left(\zeta \right){|}^{2}d\zeta \leq \kappa^{2}\;\int_{R}e^{r\zeta^{2}}{\left|w(\zeta )\right|}^{2}d\zeta \leq \kappa^{2}\;{∥w∥}_{H^{r}}^{2},$$

being $\kappa =25\;\sup_{\zeta \in R}\left\{w,D^{1}w,D^{2}w,D^{3}w,D^{4}w\right\}$.

The scaling term κ is defined according to the continuous inclusions in Sobolev spaces ([20], p. 79). Derivatives up to the third order are sufficiently regular. The fourth-order derivative is regarded as a controlling term. If this fourth-order derivative is regular, then the mollifying norm bounds the norm ${∥\cdot ∥}_{\Gamma }$.  □

Consider, now, the following representation to the homogeneous Equation (Note that the bi-laplacian is introduced as $-\Delta^{2}$ for commonality with the semi-group representation; nonetheless, in our case, the reader shall consider the one-dimensional case):

(19) $$G\left(x,t\right)=e^{-\Delta^{2}t}.$$

The operator $-\Delta^{2}$ can be seen as the infinitesimal representation of a strongly continuous semi-group with the parameter t>0:

(20) $$w\left(t\right)=e^{-\Delta^{2}t}w_{0}+\int_{0}^{t}\left[c\cdot \nabla e^{-\Delta^{2}\left(t-s\right)}w^{q}\left(s\right)+e^{-\Delta^{2}\left(t-s\right)}w\left(s\right)\left(a-w\left(s\right)\right)\right]ds.$$

Consider the Fourier transformation for $w_{t}=-w_{xxxx}$ with w(x,0)=δ(x), then:

(21) $$\tilde{w}\left(t\right)=e^{-\zeta^{4}t}{\tilde{w}}_{0}.$$

Based on this fundamental solution, the kernel for the homogeneous equation reads:

(22) $$G\left(x,t\right)=F^{-1}\left(e^{-\zeta^{4}t}\right)=\frac{1}{2\pi }\int_{R}e^{-\zeta^{4}t-i\zeta x}d\zeta =\int_{R}e^{-\zeta^{4}t}cos\left(\zeta x\right)d\zeta .$$

The last integral is bounded and exists for ζ in R.

Once a kernel has been obtained, it is possible to rewrite the abstract evolution (20) in the space $H_{\Gamma }^{4}\left(R\right)$, such that:

(23) $$T_{w_{0},t}:H_{\Gamma }^{4}\left(R\right)\to H_{\Gamma }^{4}\left(R\right),$$

given by:

(24) $$T_{w_{0},t}\left(w\right)=G\left(x,t\right)*w_{0}\left(x\right)+\int_{0}^{t}\left[c\;G_{x}\left(x,t-s\right)*w^{q}\left(s\right)+G\left(x,t-s\right)*w\left(x,s\right)\left(a-w\left(x,s\right)\right)\right]ds.$$

In the last equation, the following assessment holds for the advection:

(25) $$\begin{array}{cc} \; & G\left(x,t\right)*\;c\;{\left(w^{q}\left(x,s\right)\right)}_{x}=\int_{-\infty }^{\infty }G\left(x-\theta ,t\right)\;c\;{\left(w^{q}\left(\theta ,s\right)\right)}_{x}\;d\theta =-\int_{-\infty }^{\infty }w^{q}\left(\theta ,s\right)\;c\;G_{x}\left(x-\theta ,t\right)\;d\theta \\ & =-\int_{-\infty }^{\infty }w^{q}\left(\theta ,s\right)\;c\;\partial_{\left(x-\theta \right)}G\left(x-\theta ,t\right)\frac{\partial (x-\theta )}{\partial \theta }d\theta =\int_{-\infty }^{\infty }w^{q}\left(\theta ,s\right)\;c\;\partial_{\left(x-\theta \right)}G\left(x-\theta ,t\right)\\ & =\;c\;G_{x}\left(x,t\right)*w^{q}\left(x,t\right). \end{array}$$

The following lemma shows the bound properties of the above defined operator.

**Lemma** **2.** *The one parameter (with t) operator $T_{w_{0},t}$ is bounded in $H_{\Gamma }^{4}\left(R\right)$ with the norm *(4)*.*

**Proof.**  Firstly, the following is shown:

(26) $$b_{0}{∥w_{0}∥}_{\Gamma }\leq {∥w∥}_{\Gamma }.$$

To this end:

(27) $$\begin{array}{cc} {∥w∥}_{\Gamma }^{2} & =\int_{R}\Gamma \left(\zeta \right)\sum_{k=0}^{4}|D^{k}\hat{w}\left(\zeta \right){|}^{2}d\zeta =\int_{R}\Gamma \left(\zeta \right)\sum_{k=0}^{4}{\left|D^{k}\left[e^{t(-\zeta^{4}+q{\hat{w}}^{q-1}c\zeta i)}\;{\hat{w}}_{0}\right]\right|}^{2}d\zeta \\ & \geq \int_{R}\Gamma \left(\zeta \right)\sum_{k=0}^{4}|D^{k}\left[e^{t(-\zeta^{4}+q{\hat{w}}^{q-1}c\zeta i)}\right]{|}^{2}\sum_{k=0}^{4}|D^{k}{\hat{w}}_{0}{|}^{2}d\zeta \geq b_{0}^{2}\int_{R}\Gamma \left(\zeta \right)\sum_{k=0}^{4}{\left|D^{k}{\hat{w}}_{0}\right|}^{2}d\zeta =b_{0}^{2}{∥w_{0}∥}_{\Gamma }^{2}, \end{array}$$

such that

(28) $$b_{0}^{2}=\underset{\zeta \in B_{r}}{\inf}\{\sum_{k=0}^{4}|D^{k}\left[e^{t(-\zeta^{4}+q{\hat{w}}^{q-1}c\zeta i)}\right]{|}^{2}\}>0$$

and small in $B_{r}=\left\{\zeta ,\;\;|\zeta |<r\right\}$ for r>0.

Coming to the operator $T_{w_{0},t}$, the following holds:

(29) $$\begin{array}{cc} {∥T_{w_{0},t}\left(w\right)∥}_{\Gamma } & \leq {∥T_{w_{0},t}∥}_{\Gamma }\;{∥w∥}_{\Gamma }\leq {∥G∥}_{\Gamma }\;{∥w_{0}∥}_{\Gamma }+\int_{0}^{t}\left[{∥c\;G_{x}∥}_{\Gamma }\;{∥w^{q}∥}_{\Gamma }+{∥G∥}_{\Gamma }\;{∥w∥}_{\Gamma }\;{∥a-w∥}_{\Gamma }\right]ds\\ & \leq \left[{∥G∥}_{\Gamma }\;\frac{1}{b_{0}\;t}+\int_{0}^{t}\left[{∥c\;G_{x}∥}_{\Gamma }\;{∥w_{0}^{q-1}∥}_{H^{r}}+{∥G∥}_{\Gamma }\;\;\left|\;{∥a∥}_{\Gamma }-b_{0}{∥w_{0}∥}_{\Gamma }\right|\right]ds\right]\;\;t\;{∥w∥}_{\Gamma }. \end{array}$$

where inequalities (15) and (18) have been used for the term ${∥w^{q-1}∥}_{\Gamma }$, indeed:

(30) $${∥w^{q-1}∥}_{\Gamma }\leq {∥w^{q-1}∥}_{H^{r}}\leq {∥w_{0}^{q-1}∥}_{H^{r}}.$$

Conclusively:

(31) $${∥T_{w_{0},t}∥}_{\Gamma }\leq \left[{∥G∥}_{\Gamma }\;\frac{1}{b_{0}\;t}+\int_{0}^{t}\left[{∥c\;G_{x}∥}_{\Gamma }\;{∥w_{0}^{q-1}∥}_{H^{r}}+{∥G∥}_{\Gamma }\;\;\left|\;{∥a∥}_{\Gamma }-b_{0}{∥w_{0}∥}_{\Gamma }\right|\right]ds\right]\;\;t.$$

This last norm is bounded locally for any value in the single parameter *t*, in other words for any t>0.  □

### 2.2. Oscillating Behaviour of the Solution

The proposed analysis to show the oscillatory character of solutions is based on a shooting method approach. This technique has been previously employed in [21] for a system of equations. Nonetheless, such a process is partly modified to account for the nonlinear advection term in Equation (3).

The oscillatory behaviour is shown for a step-like initial condition:

(32) $$w_{0}\left(x\right)=H\left(-x\right),$$

*H* being the Heaviside function. The choice of a step-like function is related to the possibility of studying the asymptotic behavior in the proximity of zero as H(−x)=0 in x→∞. For the following lemma, assume preliminary that solutions exist and are unique (this will be shown afterwards) to illustrate the oscillatory behaviour of the solutions.

**Lemma** **3.** *Any solution to *(3)* with Heaviside initial condition *(32)* exhibit an oscillatory character.*

**Proof.**  We consider the following Navier conditions for |x|→∞:

(33) $$w\left(t,|x|\to \infty \right)=w^{\prime \prime }\left(t,|x|\to \infty ,\right)=0,$$

so that both derivatives are defined by the following two parameters:

(34) $$\begin{array}{c} w^{\prime }\left(t,|x|\to \infty \right)=\mu ,\;\;\;\;w^{\prime \prime \prime }\left(t,|x|\to \infty ,\right)=\omega , \end{array}$$

where μ,ω∈R.

We consider only the stationary equation to (3) as the intention is to show the oscillating solutions in view of the higher-order spatial operator:

(35) $$-w_{xxxx}+c\;{\left(w^{q}\right)}^{\prime }+w\left(a-w\right)=0.$$

A Hamiltonian holds and is of the form (refer to [14] for a complete discussion on Hamiltonians for higher-order operators):

(36) $$H\left(w\right)=w^{\prime \prime \prime }w^{\prime }-\frac{1}{2}w^{\prime \prime 2}+w^{q-1}\frac{c}{q}w^{\prime }+\frac{1}{3}w^{3}-\frac{a}{2}w^{2}+G.$$

Note that for x→∞ (in the asymptotic approximation to the stationary w=0), it can be assumed that the oscillatory character reduces, such that the action energy tends to zero, hence it can be considered that globally $w∼w^{\prime }$ and small. This approach can be followed as the idea is to study the sign character rather than the local precise behaviour of *w* and $w^{\prime }$. The Hamiltonian can be understood as an energy functional (also referred as orbit), and shall be small (null for our purposes) for |x|→∞, so that for w=0 and w=a:

(37) $$\lim_{|x|\to \infty }H\left(w^{\prime \prime \prime }\left(x\right),w^{\prime \prime }\left(x\right),w^{\prime }\left(x\right),w\left(x\right)\right)=0.$$

The value of *G* is obtained by operating in the Hamiltonian, which is made particular for one of the stationary solutions (either w=0 or w=a). For the sake of simplicity, the solution w=a is considered:

(38) $$H\left(a\right)=\frac{1}{3}a^{3}-\frac{a}{2}a^{2}+G=0\;\;\;\to G=\frac{a^{3}}{6}.$$

So that:

(39) $$H\left(w\right)=w^{\prime \prime \prime }w^{\prime }-\frac{1}{2}w^{\prime \prime 2}+w^{q-1}\frac{c}{q}w^{\prime }+\frac{1}{3}w^{3}-\frac{a}{2}w^{2}+\frac{1}{6}a^{3}.$$

Note that q>0 and might adopt a value in the interval (0,1). Then, for technical reasons, we introduce the following supporting function [18]:

(40) $$f^{\epsilon }=\frac{1}{q}{\left(w+\epsilon \right)}^{q-1}-\frac{1}{q}\epsilon^{q-1},\;\;\mathrm{with}\;\;\epsilon \to 0^{+}.$$

Any heteroclinic orbit defined between the stationary solutions w=0 and w=a can be obtained by a minimization problem in the Hamiltonian. The minimal energy of such orbits behaves as the stationary solutions for |x|→∞. Considering the Navier conditions in (33), the following holds:

(41) $$H\left(w\right)=w^{\prime \prime \prime }\left(|x|\to \infty \right)w^{\prime }\left(|x|\to \infty \right)+f^{\epsilon }cw^{\prime }\left(|x|\to \infty \right)+\frac{1}{6}a^{3}=0.$$

If $w^{\prime \prime \prime }\left(t,|x|\to \infty ,\right)=\omega$, $w^{\prime }\left(t,|x|\to \infty ,\right)=\mu$, there is a relation between both derivatives in the asymptotic approach |x|→∞:

(42) $$\mu =-\frac{a^{3}/6}{\omega +f^{\epsilon }c}.$$

According to the previous equation, the third and first derivatives have opposite signs. In addition, a solution satisfies:

(43) $$\lim_{x\to \infty }\left(w\left(x\right),w^{\prime }\left(x\right),w^{\prime \prime }\left(x\right),w^{\prime \prime \prime }\left(x\right)\right)=\left(0,0,0,0\right).$$

To prove the existence of oscillating solutions, a locating variable σ is introduced, to account for the decreasing behaviour of solutions down to w=0:

(44) $$\sigma \left(\mu \right)=\sup\left\{x>0\;\;\;|\;\;\;w^{\prime }\left(\mu ,\omega \left(\mu \right),\cdot \right)<0\;\;\;\mathrm{in}\;\;\;\left(0,x\right)\right\}.$$

Furthermore, let us define:

(45) $$\mu^{*}=\sup\{w^{\prime }\;\;\;\left|\;\;\;u\left(\mu ,\omega \left(\mu \right),\sigma \left(\mu \right)\right)<1\right\},\;\;\;\;\;\;\omega^{*}=\sup\{w^{\prime \prime \prime }\;\;\;\left|\;\;\;u\left(\mu ,\omega \left(\mu \right),\sigma \left(\mu \right)\right)<1\right\}.$$

The existence of oscillating solutions means that a finite value of σ(μ) holds. To this end, define μ as:

(46) $$\mu =-\frac{1}{\sigma (\mu )}.$$

The negative sign in the last expression permits us to state that the supreme value of μ is obtained for the supreme value of σ. Now, consider the expression (42) to state the relation between ω and μ:

(47) $$-\frac{1}{\sigma (\mu )}\omega =-\frac{a^{3}/6}{\omega +f^{\epsilon }c}.$$

We consider $\mu =\mu^{*}$ (for the supreme value of σ) and $\omega =\omega^{*}$, so that σ is given by:

(48) $$\sigma \left(\mu^{*}\right)=\frac{\omega^{*}\left(\omega^{*}+f^{\epsilon }c\right)}{a/6}.$$

Any heteroclinic orbit connecting the stationary solutions w=0 and w=1 shall exhibit a finite value of $\omega^{*}$. Indeed, the following holds:

(49) $$\lim_{x\to \infty }\left(w\left(x\right),w^{\prime }\left(x\right),w^{\prime \prime }\left(x\right),w^{\prime \prime \prime }\left(x\right)\right)=\left(0,0,0,0\right),\;\;\lim_{x\to -\infty }\left(w\left(x\right),w^{\prime }\left(x\right),w^{\prime \prime }\left(x\right),w^{\prime \prime \prime }\left(x\right)\right)=\left(a,0,0,0\right).$$

A maximum and finite value for the third derivative (referred as $\omega^{*}$) holds for any continuous non-trivial solution. Hence, $\sigma (\mu^{*})$ is finite (note that $|f^{\epsilon }|$ might be big in the proximity of the stationary solutions as per the definition given in expression (40), nonetheless it can be stated that $|f^{\epsilon }|<\infty$ in such proximity).

Operating similarly to the search for a finite spatial value $x>\sigma (\mu^{*})$, the first derivative is positive in the interval $\left(\sigma \left(\mu^{*}\right),x\right)$. To this end, define:

(50) $$\psi \left(\mu \right)=\sup\left\{\left(\;x-\sigma \left(\mu^{*}\right)\;\right)>0\;\;\;|\;\;\;w^{\prime }\left(\mu ,\omega \left(\mu \right),\cdot \right)>0\;\;\;in\;\;\;\left(\sigma \left(\mu^{*}\right),x\right)\right\}.$$

In addition:

(51) $$\mu^{**}=\inf\{w^{\prime }\;\;\;\left|\;\;\;u\left(\mu ,\omega \left(\mu \right),\psi \left(\mu \right)\right)>0\right\},\;\;\;\;\;\omega^{**}=\inf\{w^{\prime \prime \prime }\;\;\;\left|\;\;\;u\left(\mu ,\omega \left(\mu \right),\psi \left(\mu \right)\right)>0\right\}.$$

It is possible to define a suitable value of the parameter μ considering that the connecting orbit is non-decreasing in $\left(\sigma \left(\mu^{*}\right),x\right)$. As a consequence, the value of μ is positive, and according to (42), the sign of ω is negative. Considering the finite function step (δ) in the interval $\left(\sigma \left(\mu^{*}\right),x\right)$, we have:

(52) $$\mu =\frac{\delta }{\psi -\sigma },$$

Now, considering the expression (42):

(53) $$\frac{\delta }{\psi -\sigma }\omega =-\frac{a^{3}/6}{\omega +f^{\epsilon }c}.$$

Then:

(54) $$\psi =\sigma -\frac{6\delta \omega }{a^{3}}\left(\omega +f^{\epsilon }c\right).$$

Let us consider the value $\mu =\mu^{**}$, then the infimum rate of growth is obtained, leading to the supreme value of ψ. Furthermore, let us consider the infimum value of $\left(\omega^{**}\right)$. Then, the following value of ψ is the supreme of the finite spatial location:

(55) $$\psi \left(\mu^{**}\right)=\sigma +\frac{6\delta |\omega^{**}|}{a^{3}}\left(\right|\omega^{**}\left|+f^{\epsilon }c\right).$$

It has been proven that any stationary orbit between the stationary solutions w=0 and w=a is non-increasing in the interval $\left(0,\sigma \left(\mu^{*}\right)\right)$ and is non-decreasing in the interval $\left(\sigma \left(\mu^{*}\right),\psi \left(\mu^{**}\right)\right)$, where $\sigma (\mu^{*})$ and $\psi (\mu^{**})$ are finite. This monotonous behaviour of the orbits reflects the presence of instabilities (also called oscillations).  □

### 2.3. Uniqueness

The uniqueness of solutions is provided based on the definition of a map $T_{w_{0},t}$ (see (23)) that complies with a unique fix point argument, i.e. $w\left(x,t\right)=T_{w_{0},t}\left(w\left(x,t\right)\right)$. To this end:

(56) $$\begin{array}{cc} ∥ & T_{w_{0},t}\left(w_{1}\right)-T_{w_{0},t}\left(w_{2}\right){∥}_{\Gamma }\leq \int_{0}^{t}{∥c\;G_{x}\left(x,t-s\right)*\left(w_{1}^{q}-w_{2}^{q}\right)+G\left(x,t-s\right)*\left[w_{1}\left(a-w_{1}\right)-w_{2}\left(a-w_{2}\right)\right]∥}_{\Gamma }ds\\ & =\int_{0}^{t}{∥\int_{t}^{s}\left\{c\;G_{x}\left(x,t-s-r\right)\left(w_{1}^{q}-w_{2}^{q}\right)+G\left(x,t-s-r\right)\left[w_{1}\left(a-w_{1}\right)-w_{2}\left(a-w_{2}\right)\right]\right\}dr∥}_{\Gamma }ds\\ & \leq \int_{0}^{t}\int_{t}^{s}\left\{{∥c\;G_{x}\left(x,t-s-r\right)\left(w_{1}^{q}-w_{2}^{q}\right)∥}_{\Gamma }+{∥G\left(x,t-s-r\right)\left[w_{1}\left(a-w_{1}\right)-w_{2}\left(a-w_{2}\right)\right]∥}_{\Gamma }\right\}drds\\ \; & =\int_{0}^{t}\int_{t}^{s}\left\{{∥c\;G_{x}\left(x,t-s-r\right)∥}_{\Gamma }{∥w_{1}^{q}-w_{2}^{q}∥}_{\Gamma }+{∥G\left(x,t-s-r\right)∥}_{\Gamma }{∥w_{1}\left(a-w_{1}\right)-w_{2}\left(a-w_{2}\right)∥}_{\Gamma }\right\}drds\\ \; & \leq M\int_{0}^{t}\int_{t}^{s}\left\{{∥w_{1}^{q}-w_{2}^{q}∥}_{\Gamma }+{∥w_{1}\left(a-w_{1}\right)-w_{2}\left(a-w_{2}\right)∥}_{\Gamma }\right\}drds, \end{array}$$

It shall be noted that *g* and $G_{x}$ are bounded in accordance with (22). Then:

(57) $$M=\sup\{{∥{∥c\;G_{x}\left(x,t-s-r\right)∥}_{\Gamma },\;\;G\left(x,t-s-r\right)∥}_{\Gamma }\;\;\;\;\forall t>0,\;\;x\in R\},$$

for any s,r.

With the aim of assessing the resulting integrals in (56), the following function is defined:

(58) $$a\left(\epsilon ,s\right)=\left[\begin{array}{c} \frac{w_{1}{\left(\epsilon ,s\right)}^{q}-w_{2}{\left(\epsilon ,s\right)}^{q}}{w_{1}\left(\epsilon ,s\right)-w_{2}\left(\epsilon ,s\right)}\;\;for\;\;\;w_{1}≢w_{2}\\ qw_{1}^{q-1}\;\;otherwise \end{array}\right].$$

Consider two arbitrary values for ϵ and s=T; then, the previous function is bounded and satisfies the following equation:

(59) $$0\leq a\left(\epsilon ,s\right)\leq c_{0}(q,∥w_{0}{∥}_{\infty },T).$$

Then:

(60) $${∥w_{1}^{q}-w_{2}^{q}∥}_{\Gamma }\leq C_{0}{∥w_{1}-w_{2}∥}_{\Gamma },$$

where $C_{0}={∥c_{0}∥}_{\Gamma }$.

The left-hand side integral reads:

(61) $$\begin{array}{cc} \; & {∥\left[w_{1}\left(a-w_{1}\right)-w_{2}\left(a-w_{2}\right)\right]∥}_{\Gamma }^{2}=\int_{R}\Gamma \left(\omega \right)\sum_{k=0}^{4}{\left|D^{k}\left[w_{1}\left(a-w_{1}\right)-w_{2}\left(a-w_{2}\right)\right]\right|}^{2}d\omega \\ \; & =\int_{R}\Gamma \left(\omega \right)\left\{|w_{1}\left(a-w_{1}\right)-w_{2}\left(a-w_{2}\right){|}^{2}+\sum_{k=1}^{4}{\left|D^{k}\left[w_{1}\left(a-w_{1}\right)-w_{2}\left(a-w_{2}\right)\right]\right|}^{2}\right\}d\omega \\ \; & =\int_{R}\Gamma \left(\omega \right)\left\{|\left(w_{1}-w_{2}\right)(a-\left(w_{1}-w_{2}\right){|}^{2}+\sum_{k=1}^{4}\sum_{i=1}^{k}{\left|\left(\begin{array}{c} k\\ i \end{array}\right){\left(w_{1}-w_{2}\right)}^{\left(i\right)}(a-{\left(w_{1}-w_{2}\right)}^{\left(k-i\right)}\right|}^{2}\right\}d\omega \\ \; & \leq 25P^{2}\int_{R}\Gamma \left(\omega \right)\left\{|\left(w_{1}-w_{2}\right){|}^{2}+\sum_{k=1}^{4}\sum_{i=1}^{k}{\left|\left(\begin{array}{c} k\\ i \end{array}\right){\left(w_{1}-w_{2}\right)}^{\left(i\right)}\right|}^{2}\right\}d\omega \\ \; & =25P^{2}\int_{R}\Gamma \left(\omega \right)\sum_{k=0}^{4}{\left|D^{k}\left[w_{1}-w_{2}\right]\right|}^{2}d\omega =25P^{2}{∥w_{1}-w_{2}∥}_{\Gamma }^{2}. \end{array}$$

Note that $P^{2}=\max\{|a-(w_{1}-w_{2}){|}^{2},|a-\left(w_{1}-w_{2}\right){]}^{k-i}{|}^{2}\}$.

Finally:

(62) $${∥T_{w_{0},t}\left(w_{1}\right)-T_{w_{0},t}\left(w_{2}\right)∥}_{\Gamma }\leq M\left(5P+C_{0}\right)\int_{0}^{t}\int_{t}^{s}{∥w_{1}-w_{2}∥}_{\Gamma }dsdr=M\left(5P+C_{0}\right)t\left(t-s\right){∥w_{1}-w_{2}∥}_{\Gamma }.$$

For any interval with center *t* and proportionally to t−s, uniqueness holds if $w_{1}↙w_{2}$ for a contractive mapping $T_{w_{0},t}$ such that $T_{w_{0},t}\left(w_{1}\right)↙w_{1}$ in $H_{\Gamma }^{4}$.

### 2.4. Asymptotic Analysis to Determine a Local Inner Region of Positiveness

The solutions have been shown to exhibit an oscillatory behaviour. As a consequence, in the proximity of the null condition, solutions may be negative. The positiveness of solutions is explored under the following lemma:

**Lemma** **4.** *Solutions to *(3)* are positive in the inner ball region $B_{\rho (t)}$, where ρ(t) is shown to be:*

(63) $$\rho \left(t\right)=|\ln t|\;t^{1/4},$$

*for a sufficiently small t.*

**Proof.**  Firstly, assume the following scaling [11]:

(64) $$\omega =\frac{x}{t^{1/4}};\;\;\;\;\tau =\ln t\to -\infty \;,\;\;\;\;\;\;\;\;\;t\to 0^{+}.$$

The Equation (3) in the new variables and considering v(ω,τ) reads:

(65) $$v_{\tau }=\left(C-\frac{1}{4}I\right)v+v_{\omega }e^{3/4\tau }+e^{\tau }v\left(1-v\right),$$

where the operator $C=-D_{\omega }^{4}+\frac{1}{4}\omega D_{\omega }+\frac{1}{4}I$.

Consider the stationary solutions to:

(66) $$\left(C-\frac{1}{4}I\right)v_{e}=0,\;\;\;\;v_{e}\left(\infty \right)=0,\;\;\;\;v_{e}\left(-\infty \right)=1.$$

The pseudo-boundary conditions are given by the step-like Heaviside function, as pointed out in Section 2.2.

Note that the solution is expressed as the following sum:

(67) $$v\left(\omega ,\tau \right)=v_{e}\left(w\right)+\alpha \left(\omega ,\tau \right),$$

such that, closing the equilibrium with |α|≪1 and after replacement into (65), the following holds:

(68) $$\alpha_{\tau }=\left(C-\frac{1}{4}I\right)\alpha +v_{e,\omega }e^{3/4\tau }+e^{\tau }v_{e}\left(1-v_{e}\right).$$

Assume that $\alpha ,v_{e}\in H_{\Gamma }^{4}\left(R\right)\subset L_{\Gamma }^{2}\left(R\right)\subset L^{2}\left(R\right)$ and that the asymptotic smoothing permits the following separation of variables:

(69) α(ω,τ)=σ(w)ψ(τ),

where $\sigma ,\psi \in H_{\Gamma }^{4}\left(R\right)\subset L_{\Gamma }^{2}\left(R\right)\subset L^{2}\left(R\right)$.

Upon standard operations in (68):

(70) $$\frac{\psi^{\prime }}{\psi }=\frac{\left(C-\frac{1}{4}I\right)\sigma +\sigma^{\prime }e^{3/4\tau }+e^{\tau }v_{e}\left(1-v_{e}\right)\;/\;\psi }{\sigma }=K,$$

so that:

(71) $$\psi \left(\tau \right)=e^{\tau },$$

where K=1 for convenience.

Note that a solution to σ(w) is given considering the condition $v_{e}\left(\infty \right)=0$, such that:

(72) $$\left(C-\frac{1}{4}I\right)\sigma +\sigma^{\prime }e^{3/4\tau }=\sigma .$$

The operator C hosts a discrete set of eigenfunctions in $H_{\Gamma }^{4}\subset L_{\Gamma }^{2}$ [22]. Consequently, any spanned solution σ converges in $H_{\Gamma }^{4}$, and any solution can be simply expressed as:

(73) $$\sigma \left(w\right)=e^{\gamma \omega }.$$

Upon replacement in (72) and balancing the leading terms: $\gamma^{4}=-1,$ provided that

(74) $$\frac{1}{4}\omega +e^{3/4\tau }\ll 1,$$

which is equivalent to:

(75) $$t\geq \frac{\left|x\right|}{4}.$$

This is the condition to ensure that the exponential expression (73) holds. Assume, now, the following two main real roots in γ:

(76) $$\sigma_{+}=e^{\gamma \omega },\;\;\;\;\omega \to -\infty \;;\;\;\;\;\;\;\sigma_{-}=e^{-\gamma \omega },\;\;\;\;\omega \to \infty ,$$

so that:

(77) $$\alpha \left(\omega ,\tau \right)=e^{\tau }\left(e^{\gamma \omega }+e^{-\gamma \omega }\right).$$

The expression (67) reads:

(78) $$v\left(\omega ,\tau \right)=v_{e}\left(w\right)+e^{\tau }\left(e^{\gamma \omega }+e^{-\gamma \omega }\right).$$

Returning to (x,t):

(79) $$w\left(x,t\right)=v_{e}\left(\frac{x}{t^{1/4}}\right)+t\left(e^{\gamma \frac{x}{t^{1/4}}}+e^{-\gamma \frac{x}{t^{1/4}}}\right).$$

Note that |α|≪1 for x→∞, then:

(80) $$|te^{-\gamma \frac{x}{t^{1/4}}}|\ll 1\;\;\to \;\;|x|\gg \ln t\;\;t^{1/4}.$$

As lnt<0:

(81) $$\left|x\right|\ll \left|\ln t\right|t^{1/4}=\rho \left(t\right),$$

which includes the region defined in (75). Then:

(82) $$|x|<4t\ll \left|\ln t\right|t^{1/4}$$

for $t\ll 0^{+}$.  □

Finally, note that the same process can be pursued for any $t=t_{0}>0$. To this end, it suffices to assume the scaling $\tau =\ln(t-t_{0})$. Consequently, the positive region applies locally provided the following inequality holds:

(83) $$\left|x|\ll \right|\ln(t-t_{0})|{\left(t-t_{0}\right)}^{1/4}.$$

## 3. Solution Profiles

Assume the non-linear transformation:

(84) $$w=e^{u}.$$

For dedicated discussions about this proposed scaling, the reader is referred to [23]. Other interesting exploration of solutions with exponential scaling are given in [24,25,26].

As shown, the operator $\left\{\frac{\partial }{\partial t}+D_{x}^{4}\right\}$ is oscillatory; thus, any solution (or at least one of the leading profiles) shall be oscillatory. Consequently, the function *u* shall be generally defined as complex:

(85) u:X×[0,T]→C.

Following an idea in [27], the function *u* satisfies a Hamilton–Jacobi type of equation:

(86) $$u_{t}=H_{4}\left(u,\frac{\partial u}{\partial x}\right)+P_{4}\left(u,\frac{\partial^{i}u}{\partial x^{i}}\right),\;\;\;\;\;i=2,3,4,$$

where

(87) $$H_{4}\left(u\right)=-u_{x}^{2}u_{x}^{2}+cqu_{x}e^{(q-1)u}+a-e^{u},$$

and:

(88) $$P_{4}\left(u\right)=-\Delta^{2}u-\Delta \left(\nabla u\cdot \nabla u\right)-2\nabla u\cdot \nabla \Delta u-2\left(\nabla u\cdot \nabla u\right)\Delta u-2\nabla u\cdot \nabla \left(\nabla u\cdot \nabla u\right)-{\left(\Delta u\right)}^{2}.$$

For commonality with the expression used in [27], we consider the spatial derivatives with the Δ operator. The reader shall consider the one-dimensional case only. The higher-order operator $P_{4}$ is of order three, while the Hamilton–Jacobi operator is of order four. This can be shown by considering a sufficiently smooth function $\sigma \in H^{4}\left(R\right)$ with continuous derivatives and an arbitrary constant β∈R, such that:

(89) $$|P_{4}\left(\lambda \sigma \right)|=O\left(\lambda^{3}\;\partial^{i}\sigma \right)\ll H_{4}\left(\lambda \sigma \right)=O\left(\lambda^{4}\;\sigma_{x}\right),\;\;\;\;i=1,2,3,4.$$

Considering the leading terms, the Equation (3) is rewritten as:

(90) $$u_{t}=-u_{x}^{2}u_{x}^{2}+cqu_{x}e^{(q-1)u}+a-e^{u}.$$

In the search of standing wave solutions to the first-order Equation (90), we assume that such solutions are expressed making use of the standard separation of variables [27]:

(91) $$u\left(x,t\right)={\left(\tau +t\right)}^{-\frac{1}{3}}\theta \left(x\right),$$

where τ<t<T. Upon substitution in (90) and considering the asymptotic approach with t→∞:

(92) $$-\frac{1}{3}\theta =-\theta_{x}^{4}+cq\left(\tau +t\right)\theta_{x}+a_{1},$$

where it is considered τ≫1 and any $t<k_{0}\tau$, $k_{0}>1$, $a_{1}=\left(a-1\right)\;{\left(k_{0}+1\right)}^{4/3}\;\tau^{4/3}$.

Note that $e^{u}=e^{{\left(\tau +t\right)}^{-\frac{1}{3}}\theta \left(x\right)}\to 1,\;\;\;\;e^{(q-1)u}=e^{\left(q-1\right){\left(\tau +t\right)}^{-\frac{1}{3}}\theta \left(x\right)}\to 1$, for τ≫1. Making the balance of the leading terms in (92) for $|\theta_{x}|\ll 1$:

(93) $$-\frac{1}{3}\theta =cq\left(\tau +t\right)\theta_{x}+a_{1}.$$

Then:

(94) $$\theta \left(x\right)=3\left(e^{-\frac{x}{3cq(\tau +t)}}-a_{1}\right).$$

In the asymptotic approximation t→∞, the following transport front is obtained in the long spatial oscillating period (this is $|\theta_{x}|\ll 1$):

(95) $$|x|=3\;c\;q\;\ln(a_{1})\;t.$$

Balancing the first derivative $|\theta_{x}\left|\ll cq\left(\tau +t\right)\ll \right|\theta_{x}^{4}|$, we have:

(96) $$-\frac{1}{3}\theta =-\theta_{x}^{4}+a_{1}.$$

So that a solution is:

(97) $$\theta \left(x\right)=3{\left(\frac{1}{4}D\left(i\right)x\right)}^{\frac{4}{3}}-3a_{1},$$

where $D\left(i\right)={\left(-1\right)}^{\frac{1}{4}}$. As a consequence:

(98) $$u\left(x,t\right)=3t^{-\frac{1}{3}}\left({\left(\frac{1}{4}D\left(i\right)x\right)}^{\frac{4}{3}}-a_{1}\right).$$

Now and considering (84), the solution is given as:

(99) $$w\left(x,t\right)=e^{-3a_{1}t^{-\frac{1}{3}}}\;e^{3t^{-\frac{1}{3}}{\left(\frac{1}{4}D\left(i\right)x\right)}^{\frac{4}{3}}}.$$

This solution is oscillatory (according with the complex D(i)) and corresponds to the case of short spatial oscillating period, where the nonlinear term $\theta_{x}^{4}$ dominates over the single $\theta_{x}$ (which was shown to provide a propagating front when dominating).

## 4. Order Preserving and Positive Evolution

A maximal evolution is obtained in the asymptotic x→∞, where u→0. During such evolution, we search for the spatial extreme values envelope along $w^{q}$, which means ${\left(w^{q}\right)}_{x}=0$. Hence, in the asymptotic approach, the Equation (3) reduces to the homogeneous one:

(100) $$w_{t}=-w_{xxxx},\;\;\;\;w_{0}\left(x\right)\in X=L_{loc}^{2}\left(R\right)\cap L^{\infty }\left(R\right).$$

Our intention is to characterize the dynamics in the proximity of the null solution, then we will consider the step-like Heaviside initial condition $w_{0}\left(x\right)=H\left(-x\right)$.

As described, the solutions obtained for a higher-order operator exhibit oscillations. This makes the formulation of a comparison principle difficult. As a consequence, our objective is to determine the dynamics of a maximal positive and pure monotone solution for which a maximum principle holds. For this purpose, let us consider the following self-similar scaling:

(101) $$\begin{array}{c} n\left(x,t\right)=e^{t}t^{-\frac{1}{4}}f\left(y\right),\\ y=\frac{x}{t^{\frac{1}{4}}}. \end{array}$$

Introducing the expression (101) into the Equation (100), the following elliptic ODE is obtained:

(102) $$-f^{4}+\frac{1}{4}f^{\prime }y+\frac{1}{4}f=0;\;\;\;\int_{R}f\left(y\right)dy=1.$$

The following estimation for the re-scaled kernel f(y) holds [28]:

(103) $$\left|f(y)\right|\leq C_{0}F\left(y\right),\;\;\;\;F\left(y\right)=\xi_{1}e^{-k_{0}{\left|y\right|}^{\frac{4}{3}}}>0,\;\;\;\;\;\;\xi_{1}={\left(\int_{R}e^{-k_{0}{\left|y\right|}^{\alpha }}dy\right)}^{-1}.$$

**Figure 1:**
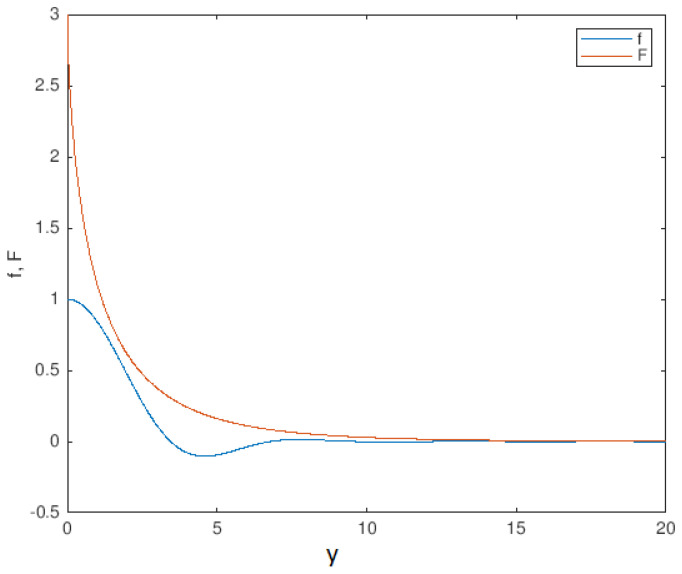
The monotone kernel *F* is kept maximal to the oscillatory kernel *f* for the for $C_{0}=3$ and $k_{0}=0.5$. The figure has been obtained by numerical explorations. Note the asymptotic similar decreasing behaviour.

The normalizing constant $\xi_{1}$ guarantees that the maximal positive kernel *F* satisfies the normalization condition $\int_{R}F\left(y\right)dy=1$, which is a necessary property to support the construction of a maximal kernel with a finite energy and in compliance with the pseudo-boundary conditions converging to zero at infinity. The parameter $C_{0}>0$ can be considered as the order deficiency of the higher order operator and shall be selected sufficiently large so that $C_{0}F\left(y\right)>f\left(y\right)$. This is particularly relevant as the decreasing rate of F(y) and f(y) are different. Figure 1 shows a combination of values for the parameters $C_{0}$ and $k_{0}$ to avoid the intersection of both functions f,F while keeping the maximal evolution of *F*.

The next step, in the characterization of the maximal kernel (*F*), is to obtain a suitable value for $k_{0}$. For this purpose, we use an asymptotic approach for the self-similar kernel elliptic ODE (102), so that:

(104) $$y\to \infty ;\;\;f\to 0\Rightarrow -f^{\left(4\right)}+\frac{1}{4}y\;f^{\prime }=0.$$

According to Figure 1, the maximal kernel F(y) behaves, asymptotically, as the solution f(y), but keeping the global monotone decreasing property. Then, $k_{0}$ can be determined by a Wentzel–Kramers–Brillouin (WKB) approximation. Asymptotically, let us consider the solution as a single parameter evolution of the form:

(105) $$e^{-k_{0}G\left(y\right)},$$

where:

(106) $$G\left(y\right)=y^{\frac{4}{3}}.$$

Now, we employ the WKB approximation into the asymptotic Equation (104), considering that:

(107) $$f^{\prime }=-k_{0}\frac{4}{3}y^{1/3}e^{-k_{0}y^{4/3}},$$

(108) $$f^{\left(4\right)}={\left(k_{0}\frac{4}{3}\right)}^{4}y^{4/3}e^{-k_{0}y^{4/3}}-{\left(k_{0}\frac{4}{3}\right)}^{3}e^{-k_{0}y^{4/3}}-\frac{2}{3}{\left(\frac{4}{3}\right)}^{2}k_{0}^{3}e^{-k_{0}y^{4/3}}-k_{0}^{3}\frac{16}{27}e^{-k_{0}y^{4/3}},$$

and into the Equation (104):

(109) $$\begin{array}{cc} \; & -{\left(k_{0}\frac{4}{3}\right)}^{4}y^{4/3}e^{-k_{0}y^{4/3}}+{\left(k_{0}\frac{4}{3}\right)}^{3}e^{-k_{0}y^{4/3}}+\frac{2}{3}{\left(\frac{4}{3}\right)}^{2}k_{0}^{3}e^{-k_{0}y^{4/3}}\\ & +k_{0}^{3}\frac{16}{27}e^{-k_{0}y^{4/3}}-\frac{1}{4}yk_{0}\frac{4}{3}y^{1/3}e^{-k_{0}y^{4/3}}=0. \end{array}$$

Balancing the leading terms:

(110) $$-{\left(k_{0}\frac{4}{3}\right)}^{4}-\frac{1}{4}k_{0}=0,\to k_{0}=Re\;\;{\left(-\frac{3^{3}}{2^{8}}\right)}^{\frac{1}{3}}.$$

Once a value for $k_{0}$ has been obtained, we note that two kernels are available at this point given by *f* and *F*:

(111) $$n\left(x,t\right)=t^{-\frac{1}{4}}e^{t}f\left(y\right),\;\;\;\;\;N\left(x,t\right)=t^{-\frac{1}{4}}e^{t}F\left(y\right),\;\;\;\;\;y=\frac{x}{t^{\frac{1}{4}}}.$$

The kernel N(x,t) represents the asymptotic evolution of the kernel n(x,t) and has the positivity property. Solutions obtained under the kernel *N* are referred as $\tilde{w}$ while solutions obtained under *n* are referred as *w*. Based on this, the following comparison lemma holds:

**Lemma** **5.** *Assume that the initial condition satisfies ${\tilde{w}}_{0}\left(x\right)\in X$, such that ${\tilde{w}}_{0}\left(x\right)\geq w_{0}\left(x\right)$, then $\tilde{w}\left(x,t\right)\geq w\left(x,t\right)$.*

**Proof.**  (112) $$\begin{array}{cc} \tilde{w}\left(t\right)-w\left(t\right) & =N\left(t\right)*{\tilde{w}}_{0}-n\left(t\right)*w_{0}\geq N\left(t\right)*{\tilde{w}}_{0}-\left|n(t)|*\right|w_{0}|\\ \; & \geq N\left(t\right)*{\tilde{w}}_{0}-N\left(t\right)*|w_{0}|=N\left(t\right)({\tilde{w}}_{0}-|w_{0}\left|\right). \end{array}$$

considering ${\tilde{w}}_{0}\geq \left|w_{0}\right|$:

(113) $$\tilde{w}\left(t\right)\geq w\left(t\right).$$

Now, consider the variable x∈R. To this end, assume a boundary problem in $R^{+}\times R^{+}$ with a symmetry at x=0 while evolving in x>0:

(114) $$w_{x}\left(t,0\right)=w_{xxx}\left(t,0\right)=0,\;\;\;\;t>0.$$

Similarly, for $\tilde{w}$:

(115) $$\begin{array}{cc} \tilde{w}\left(x\right)-u\left(x\right) & =N\left(x\right)*{\tilde{w}}_{0}\left(t\right)-n\left(x\right)*w_{0}\left(t\right)\geq N\left(x\right)*{\tilde{w}}_{0}-\left|n(x)|*\right|w_{0}|\\ \; & \geq N\left(x\right)*{\tilde{w}}_{0}-N\left(x\right)*|w_{0}|=N\left(x\right)({\tilde{w}}_{0}-|w_{0}\left|\right), \end{array}$$

so that

(116) $${\tilde{w}}_{0}\geq \left|w_{0}\right|\to \tilde{w}\left(x\right)\geq u\left(x\right),$$

as we intended to show.  □

## 5. Conclusions

The existence and uniqueness of solutions, together with an asymptotic assessment of profiles based on a Hamilton–Jacobi equation, have been analyzed for the problem in (3). The main question tracked in this analysis consisted of the exploration of positivity conditions for solutions to a higher-order operator with nonlinear advection and a KPP reaction. As a main conclusion, a maximal positive kernel has been sharply estimated as an asymptotic expansion. In addition, and under the scope of such a maximal kernel, a comparison principle has been proved to hold.

## Data Availability

Not applicable.
